# Establishment of linkage phase, using Oxford Nanopore Technologies, for preimplantation genetic testing of Coffin-Lowry syndrome with a *de novo RPS6KA3* mutation

**DOI:** 10.3389/fgene.2023.1169868

**Published:** 2023-09-14

**Authors:** Xiaojun Wen, Jing Du, Zhiming Li, Nengqing Liu, Junye Huo, Jieliang Li, Wanna Ke, Jiaqi Wu, Xiaowu Fang, Xiufeng Lin

**Affiliations:** ^**1**^ Reproductive Medicine Center, Boai Hospital of Zhongshan Affiliated to Southern Medical University, Zhongshan, China; ^**2**^ The Second School of Clinical Medicine, Southern Medical University, Guangzhou, Guangdong, China

**Keywords:** *de novo* mutation, RPS6KA3, preimplantation genetic testing, Oxford Nanopore Technologies, MARSALA, haplotype

## Abstract

**Background:** This study aimed to perform preimplantation genetic testing (PGT) for a female Coffin-Lowry Syndrome (CLS) patient with a *de novo* mutation (DNM) in RPS6KA3. It was challenging to establish the haplotype in this family because of the lack of information from affected family members. Hence, we explored a new and reliable strategy for the detection of the DNM in PGT, using Oxford Nanopore Technologies (ONT) and the MARSALA platform.

**Methods:** We performed whole-exome sequencing (WES) on the proband and confirmed the pathogenic mutation by Sanger sequencing. The proband then underwent PGT to prevent the transmission of the pathogenic mutation to her offspring. We diverged from the conventional methods and used long-read sequencing (LRS) on the ONT platform to directly detect the mutation and nearby SNPs, for construction of the haplotype in the preclinical phase of PGT. In the clinical phase of embryo diagnosis, the MARSALA method was used to detect both the SNP-based haplotype and chromosome copy number variations (CNVs), in each blastocyst. Finally, a normal embryo was selected by comparison to the haplotype of the proband and transferred into the uterus. Sanger sequencing and karyotyping were performed by amniocentesis, at 17 weeks of gestation, to confirm the accuracy of PGT.

**Results:** Using WES, we found the novel, heterozygous, pathogenic c.1496delG (p.Gly499Valfs*25) mutation of RPS6KA3 in the proband. The SNP-based haplotype that was linked to the pathogenic mutation site was successfully established in the proband, without the need for other family members to be tested with ONT. Eight blastocysts were biopsied to perform PGT and were assessed with a haplotype linkage analysis (30 SNP sites selected), to give results that were consistent with direct mutation detection using Sanger sequencing. The results of PGT showed that three of the eight blastocysts were normal, without the DNM. Moreover, the patient had a successful pregnancy, after transfer of a normal blastocyst into the uterus, and delivered a healthy baby.

**Conclusion:** The ONT platform, combined with the MARSALA method, can be used to perform PGT for DNM patients without the need for other samples as a reference.

## 1 Introduction

Coffin-Lowry Syndrome (CLS) (OMIM:303600) is a rare, X-linked intellectual disability, that is, characterized by skeletal malformations, growth retardation, hearing deficit, paroxysmal movement disorders and characteristic facial features (distance between eyes and low nasal bridge). It has a prevalence of 1:100,000–1:50,000 and occurs mainly in males, who are usually more severely affected than females (Delaunoy et al., 2006). Female heterozygotes show variable involvement, which can range from affected intelligence to quite marked facial dysmorphism, with moderate developmental delay. The syndrome is caused by mutations in RPS6KA3 (NM_004586.2), which encodes RSK2 (ribosomal S6 kinase 2). The gene is located on the short arm of chromosome X (Xp22.2) and consists of 22 exons (Song et al., 2022). More than 200 cases of CLS have been reported and approximately 70%–80% of cases arise from *de novo* mutations (DNM), which have a 50% chance of being transmitted to the offspring (Pereira et al., 2010; Cong et al., 2022). For many couples, it is an emotional burden to terminate an existing pregnancy when the fetus has been identified as carrying a mutation. Therefore, to achieve healthy pregnancies, preimplantation genetic testing for monogenic diseases (PGT-M) can be used to detect the mutations in embryos and prevent the transmission of genetic diseases from the parents.

Preimplantation genetic testing (PGT) is an effective method used to prevent the vertical transmission of monogenic disease (Theobald et al., 2020). Generally, four to ten trophectoderm cells from a blastocyst biopsy are used for the detection of mutations. The extremely limited number of initial DNA templates used for PGT-M can lead to allele dropout (ADO) during the DNA amplification process. This can result in misdiagnosis or failed diagnosis (Liu et al., 2018). To improve PGT-M accuracy, family-based haplotype linkage analysis, by single-nucleotide polymorphism (SNP) or short tandem repeats (STRs), has been applied to PGT-M and has been reported to decrease misdiagnosis rates from 3%–4% to 1.3%–1.5% (Wilton et al., 2009). Karyomapping employs analysis of single nucleotide polymorphisms (SNPs) using microarray technology to assign linked haplotypes to embryos (Handyside et al., 2010). The whole genome next-generation sequencing (NGS) is another method to detecte SNPs for karyomapping, which can identify mutated allele revealed by sequencing with aneuploidy and linkage analyses (MARSALA)(Yan et al., 2015). These methods not only enable diagnosis of the mutation in monogenic diseases but can also identify copy number variations (CNVs) and aneuploidy (Xiong et al., 2019). In addition, SNP-array data can provide genome-wide haplotyping for PGT-M. However, all of these methods require the testing of DNA samples from biological relatives, to provide information on SNPs or STRs in the linkage haplotype phase (Harton et al., 2011). Therefore, haplotype linkage analysis is not feasible for patients with DNM or where there is no proband or there is a lack of other family members.

It has been reported that there is an average of 70.3 DNMs per proband and they may cause dominant genetic disease when they occur in a specific pathogenic gene, such as RPS6KA3 (Jónsson et al., 2017). Currently, there are few systematic methods that can be used to construct the haplotype for PGT-M, when DNMs are present. Although sperm or polar bodies of the embryo have been used to establish the male or female haplotype for DNM patients, this method is tedious and expensive. For instance, multiple sperm cells are required for haplotyping, due to the low success rate of amplification from single spermatozoa, and the specific sperm that carries the disease-causing mutation must be screened (Wu et al., 2018; Yuan et al., 2020). In addition, some studies have explored the use of genetic information from parent-linked-affected embryos (Deng et al., 2021; Wang et al., 2021; Ou et al., 2022). The availability of affected embryos for this PGT-M strategy often limits its wider application.

Recently, third-generation sequencing (TGS), with a long-read length of 10 kb to several mega bases, has been performed by Nanopore (Oxford) sequencing and single-molecule real-time (SMRT) sequencing (Pacific Bioscience). It has been successfully employed to identify balanced chromosomal translocation embryos and to directly construct haplotypes in PGT-M (Cheng et al., 2021; Liu et al., 2021; Margolis et al., 2021; Pei et al., 2021). Since there have been very few reports in this area, the construction of haplotypes by TGS for DNM patients still needs further verification.

In this study, the Oxford Nanopore Technologies (ONT) was performed on *RPS6KA3* haplotype for a CLS female with a *de novo* mutation, which was a feasible method for PGT-M in patients with DNM. The MARSALA method was used to test the spouse and blastocysts. This method directly sequenced the mutation sites in RPS6KA3 and multiple consecutive nearby SNP markers, to establish the haplotype. This addressed the DNM issue for CLS in PGT-M and also provided an important reference for similar clinical cases in PGT-M.

## 2 Materials and methods

### 2.1 Patient history and ethical approval

This study describes the case of a couple who requested genetic counseling at the reproductive center of Zhongshan Boai Hospital. The proband was a 31-year-old female who was born with intellectual disability and language delay. On physical examination, she was found to have distinctive CLS features, such as distance between the eyes and low nasal bridge. However, the proband’s parents did not show any abnormalities. The couple underwent genetic counseling and whole-exome sequencing (WES). They signed a consent form that was approved by the local ethics committee. The study was approved by the ethics committee of reproductive medicine at the Zhongshan Boai Hospital.

### 2.2 Genomic DNA extraction

Genomic DNA (gDNA) was extracted from peripheral blood lymphocytes from both the couple and the proband’s parents using a DNA extraction kit (Qiagen Cat#13343, Germany), in accordance with the manufacturer’s instructions. The purified DNA concentration was measured using a Qubit^®^ 3.0 Fluorometer (Invitrogen, United States).

### 2.3 Detection of the pathogenic mutation and mutation analysis of the family

We performed WES using the proband’s DNA sample as follows: Initially, DNA was fragmented and a DNA library was prepared. The exon was then captured using the Roche KAPA HyperExome chip (Roche, Switzerland), and DNA was sequenced on the MGISEQ 2000 platform. Quality control of the sequencing data was performed as follows: The average depth of sequencing in the target region was ≥180X and the percentage of sites with an average depth of >20X in the target region was >95%. The results of the WES were aligned to hg19 Human reference genome (UCSC,http://genome.ucsc.edu/) using the BWA-MEM 2(Version 2.2.1) and duplicate sequences were removed using SAMBAMBA (Version 0.7.1). We used the snpEff (Version 4.3t) to annotate the variants. The HGMD disease database (http://www.hgmd.org), ClinVar (http://www.ncbi.nlm.nih.gov/clinvar), the 1000 Genomes Project population database (http://browser.1000genomes.org), dbSNP (http://www.ncbi.nlm.nih.gov/snp) and ExAC (http://exac.broadinstitute.org/) were used to filter the data. After filtering, the pathogenic variation was evaluated using the Intervar (Version 2.2.1). The experiment of WES and the analysis of sequencing data were performed by the Beijing Genomics Institute (BGI).

Subsequently, Sanger sequencing was used to identify candidate pathogenic variants in the proband and the proband’s parents. The primers were designed by Oligo 6.0 (http://www.oligo.net/downloads.html), F: 5′-ACT​CCT​GGG​CGA​CAG​AGT​C-3′ and R:5′-GGAAGGTCAGTAGGATAATTCAGC-3′ were used to amplify exon 17 of RPS6KA3. The forward primer was served as sequencing primer. We conducted DNA sequencing of PCR products on an ABI3500 DNA sequencer (Applied Biosystems).

### 2.4 Long-read Nanopore sequencing-based haplotype construction in RPS6KA3 core region

A total of 2 µg DNA from the proband was prepared for the ONT library preparations. Size selection of long DNA fragments was performed using the BluePippin system (Sage Science, United States), which produced DNA fragments ranging from 10kb to 60 kb. The DNA repairing and DNA end A-addition reactionswere conducted with the NEBNext FFPE Repair Mix kit (M6630, United States) and NEBNext Ultra II End Repair/dA-tailing Kit (E7546, United States). The Ligation Buffer (LNB) and Adapter Mix (AMX) in the Ligation Sequencing Kit (Oxford Nanopore Technologies SQK-LSK109, United Kingdom) was used for further ligation reactions with Quick T4 DNA Ligase in the NEBNext^®^ Companion Module (E6056, United States). A Qubit 3.0 Fluorometer was used to quantify the size of the library fragments. The prepared library is used for loading into the R9.4.1 flow cell (Oxford Nanopore Technologies, United Kingdom) with Flow CellPriming Kit (Oxford Nanopore Technologies EXP-FLP002, United Kingdom).

Sequencing was then performed on a PromethION (Oxford Nanopore Technologies, United Kingdom) running for 72 h with 100 Gb data generation. The original nanopore sequencing FAST5 files, which consist of electrical signal data, were converted to FASTQ format using Guppy_Basecaller (Version 6.0.6). The raw reads with a mean qscore template of <7 were then filtered to give the pass reads by NanoFilt (Version 2.8.0). The 50 bp in the head/tail were cut, with which parameter was NanoFilt -q 7 -l 1000 -headcrop 50 -tailcrop 50. The processed reads were aligned to the reference genomes GRCh37by Minimap2 (Version 2.22), which parameter was -ax map-ont -L -MD -Y -t 20. Then, SAMtools (v1.2) was used to transform format SAM to BAM. Structural and single point mutations were called by Longshot (Version 0.4.1), cuteSV (Version 1.0.10), sniffles (Version 1.0.12) and Pepper-Margin-Deepvariant (Version r0.7-gpu). The phasing VCF file was obtained using the BAM file as input with the following parameter - ont_ r9_ guppy5_ sup -g -phased_ Output - t 12 by Pepper-Margin-Deepvariant, and then haplotype phasing. For haplotype, the useful reads were ≥1Kb, The number of overlapping reads were ≥2, and the number of SNP sites in the reads overlapping region were ≥1. The haplotype results were sorted and visualized for the female using the likelihood-based haplotyping approach with the hidden Markov model strategy and Python (Version 3.6.0). Finally, the haplotype within 2 Mb upstream and downstream of *RPS6KA3* were screened and displayed in the report. The experiment of Nanopore sequencing and the analysis of sequencing data were performed by Yikon Genomics Company.

### 2.5 Oocyte retrieval, intracytoplasmic sperm injection and blastocyst biopsy

The standard techniques were used for *in vitro* fertilization (IVF) and the antagonist protocol was used for ovarian stimulation. The patient was treated with 150–225 IU/d of gonadotropin (recombinant human FSH, Merck Serono, Switzerland) at the beginning of day two of the menstrual cycle and the dose of gonadotropin was adjusted in accordance with ovarian reactivity. When the diameter of the follicles reached 12 mm, 0.25 mg of Nirek acetate (Merck Serono, Switzerland) was given daily. When the diameter of at least three dominant follicles reached 18 mm, 0.2 mg of triptorelin acetate (Ferring AG, Switzerland) and 4000 IU of Human Chorionic Gonadotropin (HCG, Merck Serrano, Switzerland) were administered by intramuscular injection, to trigger ovulation. After 36–38 h, transvaginal ultrasound-guided oocyte retrieval was performed.

All metaphase II (MII) oocytes were inseminated by intracytoplasmic sperm injection (ICSI). Fertilization was confirmed after 16–18 h by the presence of two pronuclei (2 PN) and the second polar body (2 PB). Embryos were cultured in G1-plus and G2-plus (Vitrolife, Sweden) sequential media (Gardner and Schoolcraft, 1999). Between four and ten trophectoderm (TE) cells were biopsied from each blastocyst on day 5 or day 6 by zona drilling with a laser, and transferred into a lysis buffer for whole-genome amplification (WGA).

### 2.6 Whole-genome amplification (WGA)

Whole-genome amplification was performed using a ChromSwift kit (Yikon, China) by Multiple annealing and looping-based amplification cycles (MALBAC), in accordance with the manufacturer’s protocol. The method was para-linear amplification, in which the 5ʹ end of the primer was a common sequence of 27 nucleotides and the 3′end was a variable eight nucleotides. The polymerase binds randomly to the DNA template and forms cyclized amplicons, after pre-amplification, in order to prevent excessive copying and ADO from being introduced into the PCR process (Liu et al., 2018).

### 2.7 The MARSALA method for SNP haplotyping and CNV analysis of each embryo

A total of 20 ng of WGA product was used for library construction by the enzyme cutting method. The 200–500 bp library was sequenced using a MGI 2000 sequencer. The average depth of sequencing was 5X. Sequencing data were analyzed for CNVs and SNP haplotyping in accordance with our in-house procedures.

The original reads were aligned and compared to the UCSC hg19 Human reference genome, to filter out the low-quality and duplicate sequences for CNV analysis. All reads were counted with a 1 Mb window unit (bin) and standardized by GC content (ratio of guanine to cytosine) and reference data set. The number of reads increased by 50% when the number of copies per bin was increased from two to three, and decreased by 50% when the number of copies per bin was reduced from two to one. The embryos with CNVs of ≥4 Mb were reported by the circular binary segmentation (CBS) algorithm. Finally, the R language program was applied to visualize the CNVs of each bin of 24 chromosomes. The sequencing data were analyzed by Yikon Genomics Company.

Using the SNP information from the whole genome of each embryo, 30 SNP markers that were located within 2 Mb upstream or downstream of the gene were selected for *RPS6KA3* SNP haplotyping. Only the SNPs that were heterozygous in one parent and homozygous in the other were considered to be informative SNPs. For example, if the husband’s SNP locus was A/, the wife’s SNP locus was AB and the embryo’s SNP locus was AB, we inferred that the embryo inherited the A allele from the husband and the B allele from the wife.

### 2.8 Embryo transfer and prenatal diagnosis

The uterine endometrium was prepared by hormone replacement therapy. After 5 days of ovulation initiated by progesterone, a normal blastocyst without pathogenic mutation or CNVs was thawed and transferred into the uterus. A clinical pregnancy was confirmed if an intrauterine gestational sac, with a fetal heartbeat, was detected by ultrasound evaluation 30–40 days after frozen embryo transfer (FET). To verify the accuracy of PGT results, amniocentesis was performed at approximately 16–24 weeks of gestation. Sanger sequencing was used to detect pathogenic mutations and the karyotype was confirmed by Chromosomal G-banding.

## 3 Results

### 3.1 Identification of a pathogenic mutation in the proband by WES, verification of the mutation by Sanger sequencing, and analysis of the pathogenic mutation, by ACMG

**FIGURE 1:**
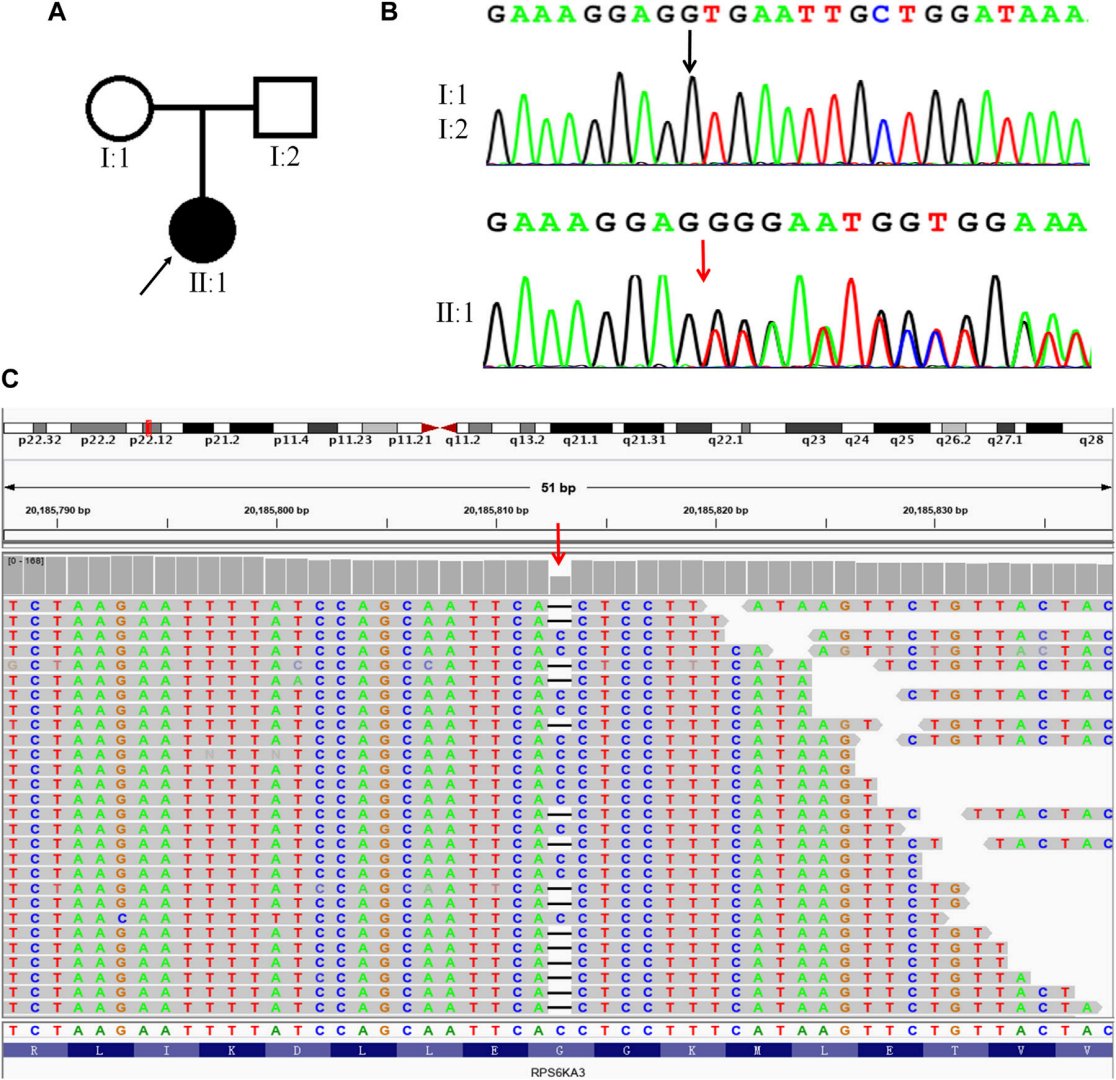
A DNM in *RPS6KA3* in a CLS female. **(A)**. Family pedigree, only the proband had CLS. A square represents males and a circle represents females. The black filled symbol represents the affected individual and the arrow points to the proband. **(B)**. Sanger sequencing of the frameshift mutation (c.1496delG) in *RPS6KA3*. The mutation (c.1496delG) was observed in II:1 but not in I:1and I:2. The black arrow points to the reference position and the red arrow points to the mutant position. **(C)** Visualizing the WES results of the proband using IGV software. The red arrow points to the mutation (c.1496delG) of the proband, horizontal line represents the base deletion.

To illuminate the genetic cause of the patient’s condition, WES analysis was performed on the proband. A total of 17,812.72 Mb of good quality data were obtained from the WES, with an average sequencing depth of 256.02%X and 99.76% coverage of the whole exome, at a depth of at least 20X. After analysis and filtering of data, a frameshift mutation (c.1496delG, p. Gly499Valfs*25) was identified in *RPS6KA3*. Visual inspection, using IGV software, confirmed that this is a true variant (Figure 1C). To determine the distribution of this mutation, we carried out Sanger sequencing on the proband and the unaffected parents. The results showed that this is a DNM in this family, with a true mutation in the proband but no mutations in the parents (Figures 1A, B).

We predicted that this *RPS6KA3* mutation causes a truncated protein, that is, easily degenerated as a result of premature termination at exon 17. The mutant site was not found in the disease (HGMD, Clinvar) and population databases (dbSNP, ExAC, 1000 genomes). The mutation was classified as a novel pathogenic mutation, which showed evidence of PVS1, PM2 and PM6, in accordance with the most recent version of the ACMG guidelines.

### 3.2 PGT for the maternal DNM in *RPS6KA3*

The clinical characteristics of the patient are shown in Table 1. Twenty oocytes were harvested for IVF treatment and intracytoplasmic sperm injection (ICSI) was performed in 16 matured oocytes (MII), whilst 15 oocytes were fertilized normally (2 PN). After sequential culture, 12 blastocysts were obtained on day 6, and eight were biopsied. The TE cells were collected from the biopsies in a 0.2 mL PCR tube filled with 5 μL of lysis buffer for later analysis. The remaining four blastocysts were frozen without being biopsied.

**TABLE 1:** Clinical characteristics of the patient.

	Patient
Female age (years)	31
Male age (years)	32
Female Karyotype	46,XX
Male Karyotype	46,XY
Female gene of mutation	*RPS6KA3*,c.1496delG (p.Gly499Valfs*25)
Length of primary infertility history(y)	1
Basal sexual hormone FSH (IU/L)	6.61
LH (IU/L)	12.73
T (nmol/L)	0.90
E_2_ (pg/mL)	52.18
AMH(ng/mL)	11.80
Protocol	GnRH antagonist
No. of oocytes retrieved	20
No. of mutured oocytes (MII)	16
No. of normal fertilization (2 PN)	15
No. of normal blastocysts	12
No. of blastocysts for biopsy	8

**FIGURE 2:**
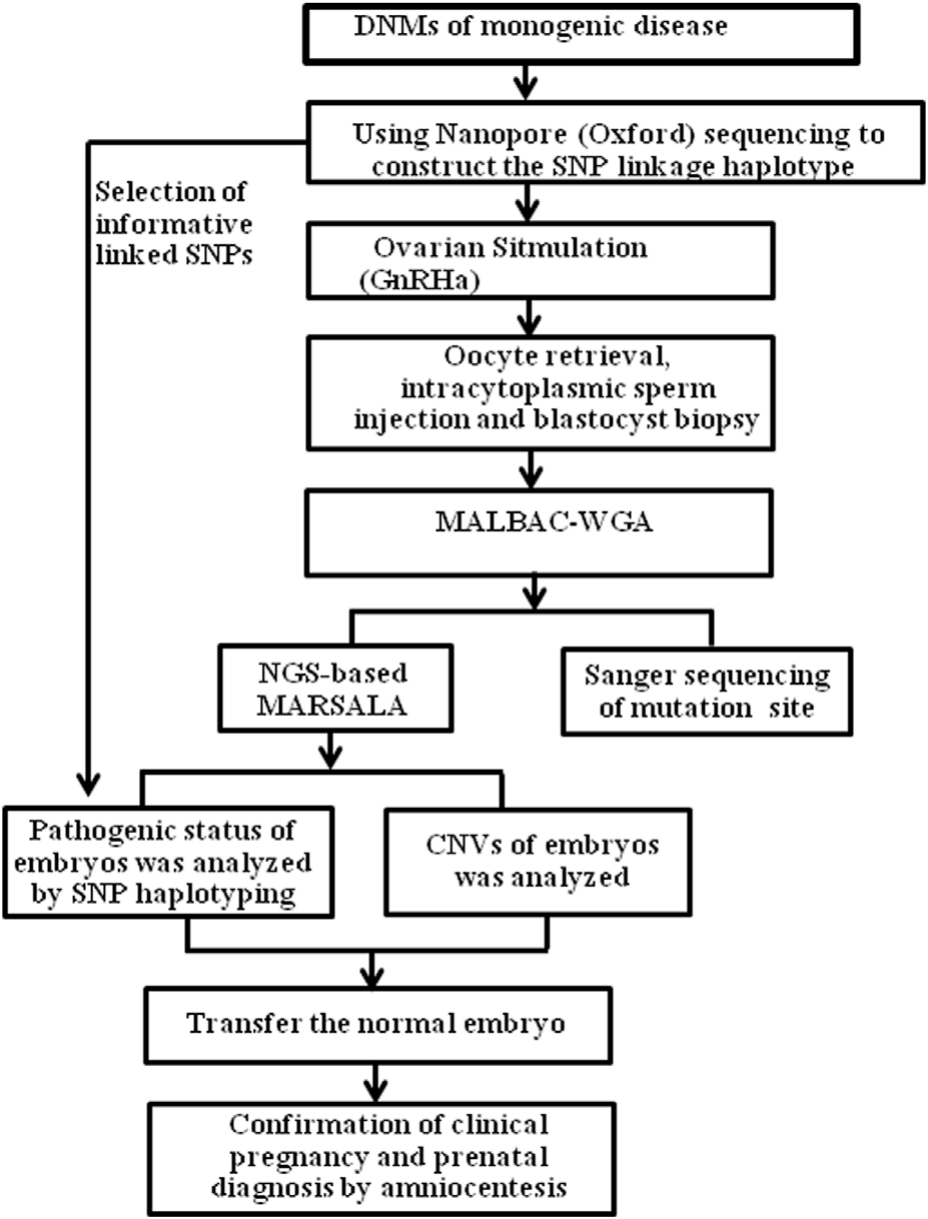
The process of SNP-based haplotyping by ONT, for PGT in patients with genetic diseases. GnRHa, gonadotrophin-releasing hormone agonist; MALBAC, multiple annealing and looping-based amplification cycles; NGS, next-generation sequencing; SNP, single-nucleotide polymorphism; CNV, Copy number variation.

It is usually difficult to construct a haplotype for maternal DNM due to the absence of the mutation in parental or nuclear family members. Using our PGT-M strategy (Figure 2), long-read sequencing enabled us to obtain long sequencing fragments (>10 kb), which contained the pathogenic mutation site and nearby SNPs. Based on ONT sequencing data, there was a sequencing depth of 28.03X, over the whole genome, and a mean read length of 14.4 kb. The linkage between the pathogenic mutation and SNP haplotype was successfully determined by long-read Nanopore sequencing.

**FIGURE 3:**
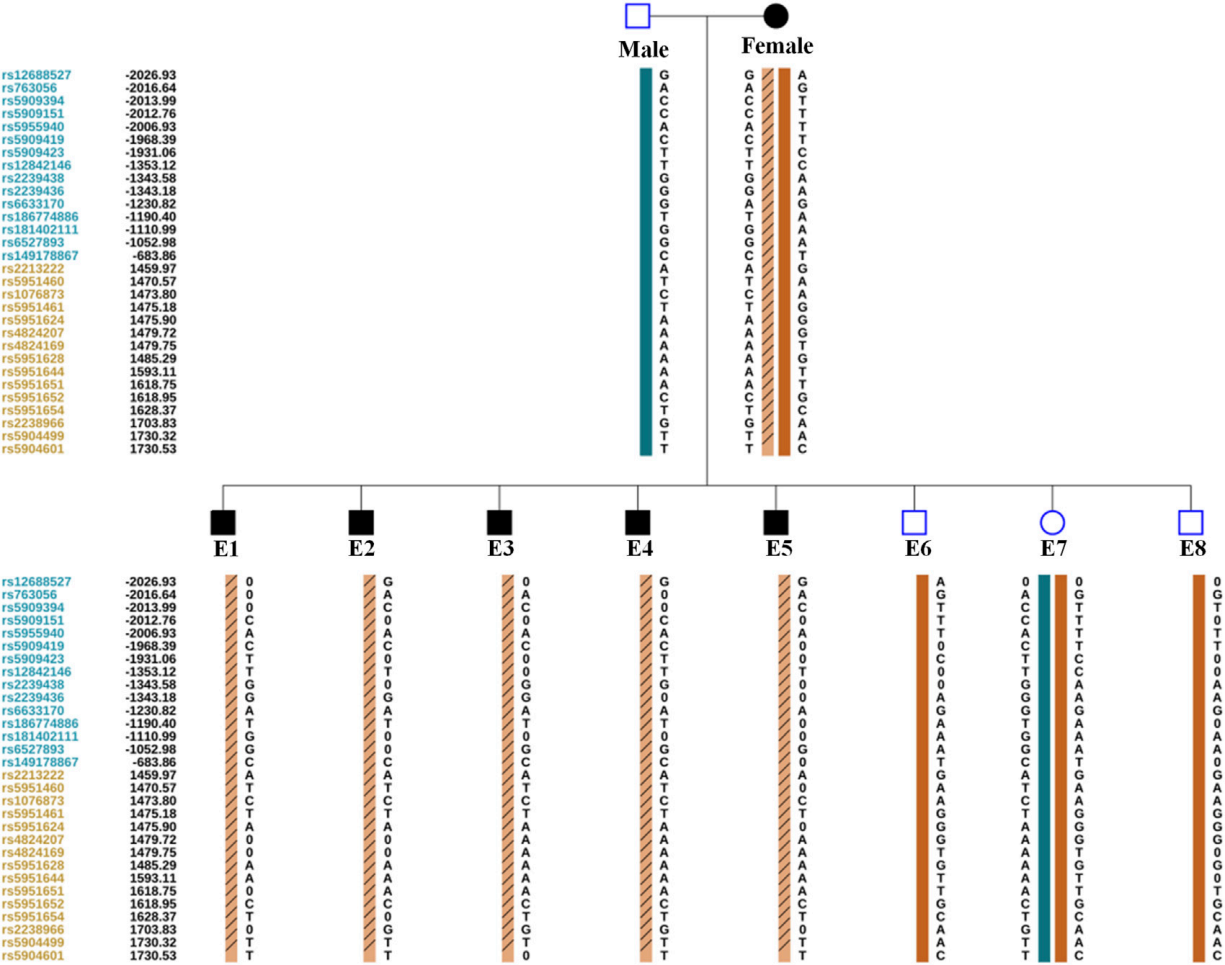
The result of PGT-M for eight embryos by haplotype analysis. A. Orange column contain slashes represent the pathogenic maternal haplotype. Orange column without slashes represents the maternal haplotype without pathogenic mutation. Green column represents the paternal haplotype without pathogenic mutation. The proband’s (Female) informative SNPs identified from Nanopore sequencing. The Male’s and embryos’ (E1–E8) informative SNPs identified from next-generation sequencing. Since male embryos have only one X chromosome, the male embryos (E1, E2, E3, E4, E5, E6, and E8) are showed only the maternal haplotype. The leftmost digits represent SNP sites, blue and orange represent SNP sites upstream or downstream of the chromosome where the gene is located. The black number on the left represents the relative distance (in Kb) from the upstream and downstream of the gene. 0 represents the missing SNP information.

**FIGURE 4:**
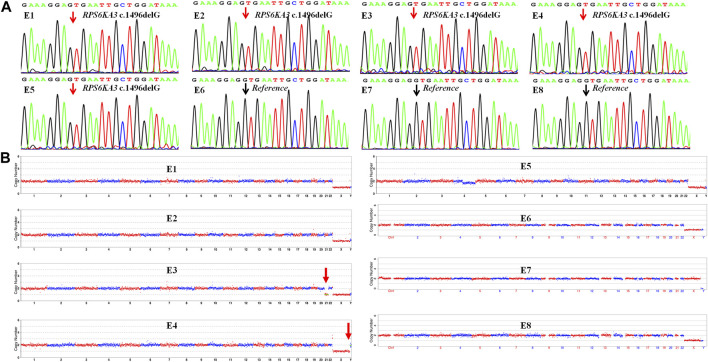
The result of CNV and verification of mutation site in each embryo (E1–E8). **(A)** Sanger sequencing to verify the mutation in each embryo, the mutation (*RPS6KA3* c.1496delG) was observed in embryos E1–E5 but not embryos E6–E8. The black arrow points to the reference position and the red arrow points to the mutant position. **(B)** Copy number variations (CNVs) of the eight embryos (E1–E8) by MARSALA. Significant chromosomal abnormality was identified in embryos E3 and E4 but not embryos E1, E2, E5, E6, E7, and E8. The red arrow points to the location of chromosomal abnormalities.

The TE cells from the eight blastocysts and the husband’s DNA were successfully used for WGA and the MARSALA method. The sequencing data had an average depth of 4.8X and Greater than 60% coverage of the target region for each embryo, and we were able to analyze the DNM in the haplotyping phase and determine CNVs (Table 2). We analyzed SNPs that were upstream and downstream of *RPS6KA3*, focusing on loci at which the female was heterozygous because the gene is located on the female X chromosome. There were 15 SNPs within 1.2 Mb upstream and 15 SNPs within 2 Mb downstream of the gene that were informative and available for haplotype analysis on the embryos. Based on haplotype linkage analysis, five of the eight embryos (E1-5) showed mutant haplotypes and were diagnosed as affected embryo. The remaining three embryos (E6-8) inherited the normal maternal haplotype and we deduced that they did not carry the maternal pathogenic mutation (Figure 3). Moreover, the Sanger sequencing results used to verify the mutation were concordant with the results of the SNP haplotype analysis (Figure 4A). To improve the chances of a clinical pregnancy and reduce the abortion rate, the MARSALA data was used to analyze CNVs in the embryos. Two of the embryos (E3, E4) showed pathogenic CNVs (Figure 4B). Among them, E4 increased the whole Y chromosome, and the karyotype was 47, XYY.

**TABLE 2:** Data from the MARSALA method, for each embryo.

Embryo	Raw_reads (M)	Raw_ bases(G)	Clean_ reads (M)	Clean_ bases (G)	Mapped ratio,%	Coverage ratio,%	Depth of average sequencing,x	Depth of average sequencing on X_chromosome,x
E1	163.37	24.51	163.27	23.53	99.95	74.00	4.80	2.50
E2	149.24	22.39	149.15	21.52	99.94	72.00	4.41	2.34
E3	139.41	20.91	139.32	20.18	99.97	72.00	4.19	2.21
E4	182.39	27.36	182.28	26.37	99.95	75.00	5.42	2.87
E5	131.57	19.74	131.50	19.04	99.95	71.00	3.93	2.04
E6	212.57	31.89	212.55	26.80	99.80	67.00	5.17	2.51
E7	255.33	38.30	255.29	31.44	99.73	68.00	5.91	5.34
E8	207.85	31.18	207.81	26.14	99.81	67.00	5.06	2.43

### 3.3 Clinical outcomes and prenatal diagnosis

**FIGURE 5:**
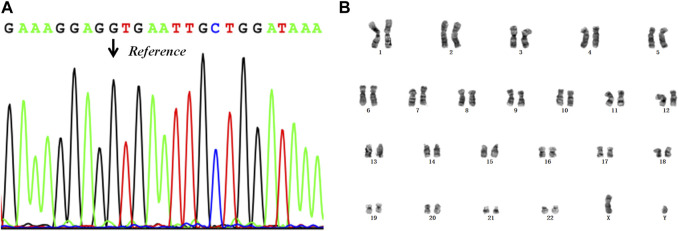
The results of the prenatal diagnosis. **(A)** Mutant detection results from the amniotic fluid sample, using Sanger sequencing. The black arrow points to the reference position. **(B)** The result of the chromosome karyotype analysis in the fetus.

According to the results of PGT, the embryos with the highest morphological score (E6, E7, E8) without pathogenic mutation and chromosomal abnormality were prioritised for transfer into the uterus. To avoid sex selection in embryos, the embryo selected for transfer was based on the order of biopsy. Therefore, a frozen embryo (E6) without the maternal mutation in *RPS6KA3* or CNVs was thawed and transplanted into the uterus of the patient (Table 3). This resulted in a pregnancy. Amniocentesis was performed at 17 weeks of gestation. The results of tests on the amniocentesis sample revealed that the fetus did not inherit the maternal mutation and the chromosome karyotype of the fetus was normal (Figure 5). A healthy baby boy who weighed 3,140 g was born at 39 weeks of gestation.

**TABLE 3:** Summary of PGT-M results for eight embryos.

Embryo	Score of embryo	The result of CNVs	Identification of maternal pathogenic mutation by haplotype	Identification of the maternal pathogenic mutation by Sanger sequencing
E1	6AA	46, XY	YES	YES
E2	5AA	46, XY	YES	YES
E3	5AA	46, XY, −21q (q11.2→q22.2,∼27 Mb,×1)	YES	YES
E4	5AA	47, XYY, +Y (×2)	YES	YES
E5	5AA	46, XY	YES	YES
E6	4AA	46, XY	NO	NO
E7	4AA	46, XX	NO	NO
E8	4AA	46, XY	NO	NO

## 4 Discussion

Coffin-Lowry Syndrome is a rare X-linked dominant disease with no currently available treatment. It is caused by a loss-of-function mutation in *RPS6KA3*. Generally, males are more severely affected than females and have the typical CLS phenotype. It is more difficult to diagnose CLS in females due to the severity of clinical features, which may be markedly variable. In this study, we used WES to successfully identify a 31-year-old female with CLS who presented with intellectual disability, language delay and coarse craniofacial features. The c.1496delG (p.Gly499Valfs*25) *RPS6KA3* mutation of the proband was classified as a *de novo* pathogenic mutation. This frameshift mutation is expected to generate a truncated protein, with loss of function, as a result of premature termination at exon 17. The mutation was described as a DNM that presented only in the proband. The parents did not have the mutation and were clinically normal. The mutation has not been reported in the disease and population databases, or in the literature. In conclusion, when combined with the clinical phenotype of the proband, c.1496delG was diagnosed as a pathogenic mutation using the classification of PVS1+PM6+PM2, in accordance with the ACMG guidelines (Richards et al., 2015).

Using a new strategy, we described the successful clinical application of long-read sequencing-based SNP haplotyping for monogenic disease with a DNM in *RPS6KA3* in PGT-M. This strategy overcomes the difficulty of requiring affected family members or embryos to construct haplotypes for the carrier couples with DNMs. In previous studies, a popular method for PGT-M has been direct mutation detection by PCR. However, due to the low amount of initial DNA from TE cells (four to ten cells), ADO is easily caused by amplification bias, which can lead to misdiagnosis or a reduced number of transferable embryos in 10%–20% of cases (Liu et al., 2018). In order to avoid misdiagnosis due to ADO, family-based linkage analysis has become a standard method to indirectly determine the mutation for PGT-M. This is achieved by identification of valid SNPs or STRs for which one parent is heterozygous and the other is homozygous. Qubbaj et al. successfully applied STR-based haplotype analysis for congenital hyperinsulinism, with homozygous mutations, in PGT-M (Qubbaj et al., 2011). Although ADO is also observed when using this method, the accuracy of PGT-M results will not be affected because of the adoption of multiple linkage marker sites for establishing haplotypes. In addition, a previous study reported that a homologous recombination phenomenon was observed in PGT-M for congenital contractural arachnodactyly. The diagnostic accuracy may be affected when using STR-based haplotype linkage analysis if the recombination occurs between STR loci and target genes. Currently, SNP-based linkage analysis is increasingly applied to PGT-M because SNPs are more widely distributed and exist at higher densities in the genome than STRs (Wang et al., 2017).

The method of haplotype analysis described above was performed with DNA from probands or nuclear family members to establish linkages between the mutated genes and informative markers. In this study, maternal linkage analysis was difficult due to the lack of information from the patient’s lineal relatives. At present, several methods have been applied to solve the problem of constructing haplotypes for DNM patients in PGT-M. These methods can be divided into two categories. The first is single-sperm-based SNP haplotyping, in which a single sperm cell is isolated and SNP genotypes are analyzed to establish the haplotype of individual sperm using NGS. Although sperm samples can be easily obtained, this technology is limited in application because of the tedious steps involved, the high cost and its suitability for only paternally-inherited dominant diseases. For females with DNM, polar bodies can be taken from probands to construct a haplotype. However, this approach also has obvious drawbacks. For example, it is difficult to successfully construct a haplotype if the patient has a poor ovarian reserve, with insufficiently mature oocytes (Wang et al., 2017; Chen et al., 2019; Yuan et al., 2020; Huang et al., 2022). The second category uses affected embryos as probands in the linkage phase and analyzes them via NGS. A previous study by Li et al. (2020) successfully applied this strategy in PGT-M for alpha thalassemia and Norrie disease protein (NDP) gene disorder, without recruiting additional family members. Ou et al. (2022) successfully performed PGT-M using affected embryos as a reference in 36 couples with thalassemia to reveal the feasibility of this approach. The limitation of this method is that it may not be possible to obtain sufficient affected embryos as a reference.

In recent years, long-read sequencing technology (LRS), performed by PacBio SMRT and ONT, has started a new era of sequencing. It can provide advantages over traditional short read sequencing-based NGS as it produces reads of approximately 10 kb or longer and can be applied to the detection of complex structural variation, insertions and deletions of <50 bp and single-nucleotide variants (Wenger et al., 2019). In addition, the haplotype between a mutation and the upstream or downstream SNPs can be obtained directly by LRS, without the need for samples from additional family members. There have been a few studies that have used LRS for haplotype construction in PGT-M. The SMRT technology was used by Wilbe et al. (2017) for haplotype construction in two families. Construction of the haplotype failed in one family, which may have been due to the short amplicon fragments that were sequenced (3.8 and 3.9 kb in the two families). Although Wu et al. (2022) successfully used SMRT for haplotype construction in three families with thalassemia, in PGT-M, the length of the amplified sequences that contained the pathogenic gene were less than 10 kb, which may lead to insufficient SNP information to analyze the haplotype.

In this study, a novel approach was applied to a PGT-M case with DNM. That is, the DNA of a proband with DNM was sequenced using ONT, to directly obtain the linkage analysis haplotype from long-read DNA sequences, which contained the mutation site and nearby SNPs. Here, we successfully constructed the haplotype of *RPS6KA3* in the proband by analyzing ONT sequencing data with an average read length of approximately 14.4 kb and an average sequencing depth of 28.03X. Therefore, the ONT platform may enable us to obtain more SNP information for haplotype construction because of the longer sequence fragment length, which reduces the failure rate of haplotype construction. We also adopted a method for WGA, using MALBAC, which has been demonstrated to have high reproducibility and good coverage. We combined it with the universal MARSALA method to obtain the SNP information for haplotyping and determine mutations in the embryos. The MARSALA data showed approximately 5X sequencing depth on the whole genome and 3X sequencing depth on the X chromosome, for each embryo. These data can be used to analyze CNVs and aneuploidy. Approximately 30 effective SNP markers, within 2 Mb upstream and downstream of the mutation site, were used to establish the haplotype. Finally, to verify the accuracy of the PGT-M results, we used Sanger sequencing to directly detect *RPS6KA3* c.1496delG mutation in each embryo. Using the haplotype linkage analysis and Sanger sequencing, we showed that five out of eight blastocysts inherited the mutation from the proband (E1, E2, E3, E4, and E5), whilst the remaining three embryos did not carry the pathogenic mutation and the results of the CNV analysis were normal.

In summary, this study is the first report of a *de novo RPS6KA3* c.1496delG mutation in a female patient with CLS. This expands *RPS6KA3* mutation spectrum and also provides a new strategy based on ONT and MARSALA to perform PGT-M with DNMs, in which constructing the haplotype of pathogenic gene of the female patient is without need for reference pedigree samples. This removes the major limitation of the single-sperm-based, polar body-based and affected embryo-based SNP haplotype analyses. Our strategy is cheaper and has been demonstrated to be a reliable and simplified PGT-M procedure for DNM patients as it reduces the amount of work involved in the amplification of multiple sperm or the collection of polar bodies. Our study provides a reference for the application of ONT and MARSALA to the PGT-M of other DNM monogenic diseases. However, this strategy has only been applied to one case and more cases are needed to prove the feasibility of this strategy for PGT-M.

## 5 Conclusion

The DNM (c.1496delG) in *RPS6KA3* can lead to CLS. The ONT method, combined with the MARSALA method, can be used to perform PGT-M, without the need for other samples as a reference for the CLS patient with a DNM. In addition, this strategy may also be used to conduct PGT-M for other monogenic diseases with DNMs.

## Data Availability

The datasets presented in this study can be found in online repositories. The names of the repository/repositories and accession number(s) can be found below: CNGB Sequence Archive (CNSA) of China National GeneBank DataBase (CNGBdb) https://db.cngb.org/search/project/CNP0004607/ with accession number CNP0004607.
